# 多发性骨髓瘤疗效评估与微小残留病监测中国指南（2024年版）

**DOI:** 10.3760/cma.j.cn121090-20241111-00444

**Published:** 2024-12

**Authors:** 中国临床肿瘤学会（CSCO）骨髓瘤专家委员会 中国抗癌协会（CACA）血液肿瘤专业委员会骨髓瘤与浆细胞疾病学组

**Keywords:** 多发性骨髓瘤, 疗效评估, 微小残留病, 指南, Multiple myeloma, Response assessment, Minimal residual disease, Guideline

## Abstract

在多发性骨髓瘤（MM）治疗过程中，应用统一的标准对疗效进行评价对于指导治疗、判断预后及解读不同研究结果非常重要。因为疾病本身的特性，MM的疗效判断较其他肿瘤更为复杂，主要基于血、尿单克隆免疫球蛋白（M蛋白）的测定，结合骨髓中浆细胞数量及全身影像学。近年来，随着新药的不断涌现，MM疗效显著提高，微小残留病检测能够显示更深层次的缓解，其临床意义目前已取得广泛共识。在此基础上，中国临床肿瘤学会（CSCO）骨髓瘤专家委员会和中国抗癌协会（CACA）血液肿瘤专业委员会组织相关专家制定了本指南，旨在为MM的疗效评估提供全面参考。

## Abstract (en)

The application of standardized criteria to evaluate treatment efficacy is crucial for guiding therapeutic decisions, assessing prognosis, and interpreting outcomes in treating multiple myeloma（MM）. The unique characteristics of MM make its response assessment more complex compared with other malignancies. It primarily depends on serum and urine M protein levels, combined with the number of plasma cells in the bone marrow and comprehensive imaging findings. Recently, the emergence of novel therapeutics has led to significant improvements in MM outcomes. Minimal residual disease（MRD）detection determines deeper remission states. The clinical significance of MRD monitoring is now widely recognized. The Chinese Society of Clinical Oncology（CSCO）MM Expert Committee and the Hematologic Oncology Committee of the Chinese Anti-Cancer Association（CACA）have gathered relevant experts to develop the guidelines based on these advancements, aiming to provide a comprehensive reference for response assessment in MM.

准确的疗效评估对于多发性骨髓瘤（MM）调整治疗方案和判断预后均至关重要。骨髓瘤细胞灶性分布的特点给MM的疗效评估带来了一定难度。传统疗效评估主要依据血清和尿液中的M蛋白水平、骨髓中的浆细胞比例及髓外浆细胞瘤的大小。随着新药的应用，越来越多的患者能够达到深度缓解。因此，微小残留病（MRD）检测在MM疗效评估中的地位愈加重要[1]。多参数流式细胞术（MFC）、新一代测序（NGS）、PET-CT等技术均为检测MRD的良好手段，然而上述技术均有局限性，需要进一步标准化。MRD检测方法及其结果对治疗的指导意义尚需进一步确认。当前，国内各临床中心疗效评估尚不规范。为解决这一问题，中国临床肿瘤学会（CSCO）骨髓瘤专家委员会和中国抗癌协会（CACA）血液肿瘤专业委员会组织相关专家制定了本指南。

一、传统疗效评估体系

当前MM的疗效评估主要遵循国际骨髓瘤工作组（IMWG）的标准。

（一）检测内容

1. 血液和尿液中M蛋白相关检测：血清/尿蛋白电泳（PEP）、血/尿免疫固定电泳（IFE）、血清游离轻链、24 h尿M蛋白定量。上述检测方法均能用于检测M蛋白，且各有优缺点，在临床应用中建议综合使用上述方法进行M蛋白的定性和定量分析（表1）。

**表1:** 国际骨髓瘤工作组推荐的多发性骨髓瘤疗效评估相关监测指标

指标		每个疗效评估时间点	当PEP检测不到M蛋白	预估达CR	疑似PD（临床或生化）
血清PEP（血M蛋白≥10 g/L）^a^		√	不适用	√	√
血清IFE		×	√	√	√
尿PEP（尿M蛋白≥200 mg/24 h）		√	不适用	√	√
尿IFE		×	√	√	×
sFLC	血、尿M蛋白均不可测，rFLC异常，并且受累轻链≥100 mg/L	√	×	√	√
	其他情况	×	×	√	√
骨髓涂片/活检	血、尿M蛋白和sFLC均不可测量，基线骨髓浆细胞比例≥30％	√（每3～4个周期复查，直到疾病平台期，或者根据临床情况检查）	×	√	√
	其他情况	×	×	√	×
浆细胞瘤（PET-CT）	如果血、尿M蛋白和骨髓浆细胞均未达到可测量标准，且存在至少1个直径≥2 cm病灶	√（每3～4个周期复查，直到疾病平台期或CR，或者根据临床情况复查）	×	√	√
	其他情况	×	×	√	×
HGB、血钙、肌酐等		√	×	×	√

**注** √：需要进行检查；×：不需要进行检查；PEP：蛋白电泳；IFE：免疫固定电泳；sFLC：血清游离轻链；rFLC：血清游离轻链比值；PET-CT：正电子发射断层显像计算机体层摄影术；CR：完全缓解；PD：疾病进展；^a^如果测定的缓解终点为≥非常好的部分缓解，且无进展生存期和疾病进展时间是主要研究终点，则基线M蛋白≥5 g/L是可接受的

2. MM肿瘤细胞数量相关检测：在MM疗效评估中，对于可能达到完全缓解（CR）的患者需进行骨髓检查。如果患者同时进行骨髓涂片和骨髓活检，应以两者中较高的数值确定骨髓中浆细胞的比例。利用免疫组化技术、流式细胞术及NGS可判断浆细胞是否为克隆性。

3. 影像学检查：包括PET-CT、CT和MRI扫描，可测量肿块大小，计算病灶最大垂直直径乘积之和（SPD）。对于有皮肤受累的患者，可直接用尺子测量皮肤肿块的大小。

（二）检测时间点

诱导治疗期间，推荐每周期评估疗效。启动新治疗后第1年建议每周期评估疗效，之后每2个周期评估疗效。血、尿M蛋白均可测量的患者，每次疗效评估均需对两者进行检测；只有一项可测量的患者，后续疗效评估中仅需检测相应M蛋白。对于血或尿M蛋白可测量的患者，不需要常规检测血清游离轻链。除非是仅有骨髓浆细胞作为可测量指标的不分泌患者，否则只需要在确认是否达到CR时复查骨髓。疗效评估不需要影像学复查骨骼，但临床实践中推荐每年复查一次。

（三）评估过程

1. 确定评估指标：可测量指标的定义是满足以下任一条件：血清M蛋白浓度≥10 g/L；尿M蛋白≥200 mg/24 h；血清游离轻链比值异常，且受累轻链浓度≥100 mg/L。若血清M蛋白和尿M蛋白均可测量，则应同时使用这两个指标进行疗效评估，疗效的判断以两者中改善程度较低者为准。只有一项可测量的患者，后续疗效评估中仅根据相应M蛋白评估，但需确定是否达到非常好的部分缓解（VGPR）或CR时，要求两者均符合相应要求。只有在血清M蛋白和尿M蛋白均无法评估的情况下才用血清游离轻链差值进行评估。如果上述三个指标均无法测量，但基线时浆细胞比例≥30％，可以根据浆细胞比例的变化评估疗效。对于有可测量指标的患者，在评估疗效的过程中无需考虑骨髓浆细胞比例，除非在判断是否达到CR时。如果基线时存在长径≥2 cm的浆细胞瘤，后续每次疗效评估中均需监测浆细胞瘤的状况，但无需在每个治疗周期进行影像学检查，通常每2～4个疗程评估一次即可。

2. 疗效评估的具体内容：见表2。

**表2:** 国际骨髓瘤工作组多发性骨髓瘤（MM）疗效评估标准

疗效	标准
完全缓解（CR）	①血清和尿免疫固定电泳阴性，软组织浆细胞瘤消失，骨髓中浆细胞<5％； ②对于仅依靠sFLC水平作为可测量指标的患者，除满足以上CR标准外，还要求sFLC比值恢复正常（0.26～1.65）
严格意义的CR（sCR）	满足CR标准的基础上要求sFLC比值正常及经免疫组化检测证实骨髓中无克隆性浆细胞（针对轻链为κ型或λ型患者，计数≥100个浆细胞，κ/λ比值≤4∶1或≥1∶2）
非常好的部分缓解（VGPR）	①蛋白电泳检测不到M蛋白，但血清和尿免疫固定电泳阳性； ②或血清M蛋白降低≥90％且尿M蛋白<100 mg/24 h； ③对于可测量指标仅有sFLC水平的患者，除满足以上VGPR标准外，还要求受累和未受累sFLC的差值缩小≥90％； ④除上述标准外，若基线存在软组织浆细胞瘤，则要求浆细胞瘤SPD缩小≥90％
部分缓解（PR）	①血清M蛋白减少≥50％，同时24 h尿M蛋白减少≥90％或降至<200 mg/24 h； ②若血清和尿中M蛋白无法检测，则要求受累与非受累sFLC的差值缩小≥50％； ③若血清和尿中M蛋白及sFLC均不可测定，且基线骨髓浆细胞比例≥30％时，则要求骨髓内浆细胞数目减少≥50％； ④除上述标准外，若基线存在软组织浆细胞瘤，则要求浆细胞瘤SPD缩小≥50％
微小缓解（MR）	①血清M蛋白减少25％～49％，同时24 h尿M蛋白减少50％～89％； ②除上述标准外，若基线存在软组织浆细胞瘤，则要求浆细胞瘤缩小25％～49％； ③溶骨性病变数量和大小未增加（可允许压缩性骨折发生）
疾病稳定（SD）	不符合CR、VGPR、PR、MR及PD标准。如做影像学检查，则应无新的骨质病变或原有骨质病变进展的证据
疾病进展（PD）	诊断至少应符合以下1项（以下数据均为与获得的最低值相比）： ①血清M蛋白升高≥25％（且升高绝对值需≥5 g/L），若基线血清M蛋白≥50 g/L，M蛋白增加≥10 g/L即可； ②尿M蛋白升高≥25％（且升高绝对值需≥200 mg/24 h）； ③若血清和尿M蛋白无法检出，则要求受累与非受累sFLC的差值增加≥25％（且增加绝对值>100 mg/L）； ④若血清和尿M蛋白及sFLC均不可测定，则要求骨髓浆细胞比例升高≥25％（且增加绝对值≥10％）； ⑤出现新的软组织浆细胞瘤病变；或原有1个以上的可测量病变SPD较最低点增加≥50％；或原有的横轴≥1 cm病变的长轴增加≥50％； ⑥循环浆细胞增加≥50％（在可测量指标仅有循环浆细胞时应用，绝对值至少为200个细胞/µl）
临床复发	符合以下1项或多项： ①出现新的骨病变或软组织浆细胞瘤（骨质疏松性骨折除外）； ②明确的已有的浆细胞瘤或骨病变增加（可测量病变SPD增加50％且绝对值≥1 cm）； ③高钙血症（血钙>2.75 mmol/L）； ④HGB下降≥20 g/L（与治疗和非MM因素无关）； ⑤自MM治疗开始，血肌酐上升≥2 mg/dl，且与MM相关； ⑥血清M蛋白相关的高黏滞血症
CR后复发（只有研究终点是无病生存期时才使用）	符合以下1项： ①免疫固定电泳或蛋白电泳证实血或尿M蛋白再次出现； ②骨髓浆细胞比例≥5％； ③出现以上PD标准中至少1项

**注** 所有疗效类别在开始新治疗前都需要连续两次评估，间隔时间不限。骨髓检查不需要重复。判断治疗有效对影像学检查不作要求，但如果进行了影像学检查，需要没有骨病进展或新发损害的证据。sFLC：血清游离轻链；SPD：病灶最大垂直直径乘积之和

3. 传统疗效评估注意事项

（1）在判断治疗效果时，应选择距开始治疗时间最近的时间点作为基准。判断疾病是否进展时，则应选择治疗后达到最佳疗效的时间点作为基准。若血清或尿液的峰值浓度过低，无法进行准确测量，应被记录为0，作为跟踪疾病后续进展的基准。

（2）评估部分缓解（PR）或微小缓解（MR），则患者必须在研究开始时（即基线时）有可测量的指标。对于基线时没有可测量指标的患者，仅评估是否达到CR或是否进展。

（3）在IgA型或IgD型MM中，也可使用免疫比浊法检测IgA/IgD的含量，以此替代血清M蛋白的测定，但必须确保检测前后的方法保持一致。对于初诊时显示出两条单克隆蛋白带的患者，应通过计算两个峰的总和确定M蛋白的量。

（4）24 h尿M蛋白检测是疗效评估的重要组成部分。不推荐用随机或24 h尿液样本检测Kappa和Lambda轻链水平替代标准的24 h尿M蛋白检测。

（5）基线时记录的软组织浆细胞瘤需要持续监测，如果不进行监测，患者将被视为无法评估。

（6）对于表现为完整球蛋白型的MM，若发现血清游离轻链显著升高，应注意是否发生了表现为轻链逃逸的疾病进展[2]。

（7）为减少疗效评估的误差，应在调整治疗方案前对除骨髓检查、影像学外的其他指标进行至少两次连续评估，两次评估的具体间隔时间无特殊要求。需要获取两份独立样本，测试结果不能仅基于单个样本的拆分。如果只有一次评估结果，该评估应被视为“不确定”。对于疗效较前次下降但尚未达到疾病进展的患者，应继续处于已确认的最佳缓解状态，而不应转移至较低的缓解类别。在评估过程中，如果有多个指标可以评估缓解程度，应选择最低缓解程度的指标作为标准。

（8）对于之前被判定为已达到CR的患者，仅凭IFE阳性结果不应判断为疾病进展，应该判断为缓解后复发。在计算至疾病进展时间（TTP）和无进展生存（PFS）期时，已达到CR且MRD检测结果阴性的患者也应按照疾病进展的标准进行。另外，针对CR后复发或MRD复发，其标准仅适用于计算无病生存（DFS）期。

（9）如果血清或尿IFE结果显示与既往不同的轻链和（或）重链阳性结果，需注意排除免疫重建寡克隆条带的可能，注意与疾病复发鉴别。此外还要注意治疗性单抗的影响[2]–[3]。

（10）除非怀疑疾病进展，在评估疗效时不需要常规进行骨骼检查。

三、MRD检测的应用

（一）MRD检测方法

目前用于检测MRD的技术包括MFC、NGS、影像学技术、蛋白质谱技术（MS）等，上述技术各有优缺点。

1. MFC：MFC在MRD检测领域应用广泛。欧洲EuroFlow联盟已对新一代MFC（NGF）进行了标准化处理，采用了一种经典方案，第一管包括CD45/CD138/CD38/CD56/CD19/CD27/CyIgκ/CyIgλ，第二管包括CD45/CD138/CD38/CD56/CD19/CD27/CD117/CD81。每个检测至少获取5×10^6^个细胞，总共至少获取10^7^个细胞，以确保检测的灵敏度达到2×10^−6^［4]。值得注意的是，使用多表位抗体针对CD38可以避免在使用CD38单克隆抗体治疗后出现假阴性结果。MSKCC采用了一种包含10个参数的单管方案，该方案包括CD45/CD138/CD38/CD56/CD19/CD27/CD117/CD81/CyIgκ/CyIgλ，至少采集3×10^6^个细胞，使得检测的灵敏度达到了6×10^−6^[5]–[6]。推荐将MFC作为当前MRD检测的常规方法，并将MRD阴性定义为瘤细胞比例小于1×10^−5^[7]–[8]。

2. NGS：NGS通过对浆细胞Ig基因重排VDJ片段的识别和量化来实现，需以患者治疗前的标本作为对照。NGS MRD检测的灵敏度在复查时与送检的DNA量有关，在送检20 µgDNA的情况下（相当于约3×10^6^个有核细胞），NGS检测MRD的灵敏度可达1×10^−6^[9]–[11]。多项研究表明，在同等灵敏度下，NGF与NGS的检测结果一致性超过80％[4],[12]。

3. 影像学：影像学的价值主要表现在两个方面：首先，能够协助评估髓外浆细胞瘤的情况。其次，影像学的缓解是对骨髓MRD结果的有益补充。PET-CT是检测MRD相关研究中使用最多的影像学方法。确定基线PET-CT阳性病灶的标准是骨内存在局灶性摄取增高区域，无论是否伴有CT发现的任何潜在病灶，且病灶至少连续出现在两个层面上。一旦通过NGF或NGS检测到MRD阴性，应进行影像学检查。多项研究显示，如果骨髓和PET-CT检测均显示MRD阴性，患者的预后最佳[13]–[17]。

（二）MRD评估的具体内容

MRD评估的具体内容见表3。

**表3:** 国际骨髓瘤工作组推荐的多发性骨髓瘤（MM）微小残留病（MRD）评估标准

疗效	标准
持续MRD阴性	新一代流式细胞术（NGF）和（或）新一代测序（NGS）检测骨髓MRD阴性且影像学检查阴性，至少间隔1年，两次检测均为阴性。进一步评估用MRD阴性持续时间描述，如“5年MRD阴性”
流式细胞术MRD阴性	NGF检测显示骨髓无表型异常的克隆性浆细胞，流式细胞术采用EuroFlow标准操作规程（或应用经过验证的等效方法），最低检测敏感度为10^5^个有核细胞中可检测到1个克隆性浆细胞
测序MRD阴性	采用NGS深度测序方法（Lympho SIGHT平台或经过验证的等效方法）检测患者骨髓中无克隆性浆细胞（定义为同样的测序读长少于2个）。最低检测敏感度为10^5^个有核细胞中可检测到1个克隆性浆细胞
原有影像学阳性的MRD阴性	要求NGF或NGS检测MRD阴性，且原有PET-CT上所有高代谢病灶消失，或病灶标准摄取值（SUV）低于纵隔血池，或低于周围正常组织的SUV值
MRD阴性后复发（仅用于临床研究）	符合以下任意一项或多项标准： 失去MRD阴性状态（NGF或NGS证实存在克隆性浆细胞，或影像学提示MM复发）；免疫固定电泳或蛋白电泳检测血清或尿中M蛋白再次出现；骨髓中克隆性浆细胞≥5％；出现任何其他疾病进展情况（如新的浆细胞瘤、溶骨性破坏或高钙血症）

（三）MRD评估的注意事项

1. 样本采集：针对骨髓NGF标本，建议使用抽吸的第一管标本进行送检。样本至少2 ml以确保采集到足够的细胞，但样本量不应超过4～5 ml，以免过度稀释。若第一管标本难以获得，建议调整穿刺位置再取。MFC可以通过观察有核细胞成分的分布评估标本质量。

2. 检测手段：较高灵敏度（10^−5^或10^−6^）检测方法判断的MRD阴性者预后优于较低灵敏度检测方法判断的MRD阴性者[18]–[19]。如果报告持续的MRD阴性状态，应该注明使用的方法（如持续NGF MRD阴性、持续NGS MRD阴性）。

3. 检测时间点：目前，MRD检测时间尚无统一标准。处于CR状态的患者进行MRD检测，能够提供最佳的预后价值。推荐在诱导治疗后、自体造血干细胞移植后3个月、巩固治疗后及维持治疗期间进行MRD检测。

4. MRD临床指导意义：MRD阴性是MM最重要的动态预后指标[20]。2024年FDA肿瘤药物咨询委员会（ODAC）会议决定将MRD作为MM临床试验加速批准的替代终点。然而MM目前仍被视为不可治愈的疾病，MRD阴性并不能阻止疾病复发。关于如何利用MRD指导MM治疗，目前尚无明确的推荐或共识。

在MM治疗过程中，运用统一的标准评价疗效对于指导治疗、判断预后及解读不同研究的结果均极为重要。由于疾病本身的特性，MM的疗效判断较其他肿瘤更为复杂。常规的疗效评估主要基于血清和尿液中M蛋白的测定，并结合骨髓中浆细胞数量及全身影像学情况，上述因素共同构成了MM疗效判断的基础。尽管MRD的预后意义已被广泛认可，但其在临床上的具体指导作用尚无明确共识。
